# Incidence estimation from sentinel surveillance data; a simulation study and application to data from the Belgian laboratory sentinel surveillance

**DOI:** 10.1186/s12889-019-7279-y

**Published:** 2019-07-23

**Authors:** Toon Braeye, Sophie Quoilin, Niel Hens

**Affiliations:** 1 Epidemiology of Infectious Diseases, Epidemiology, Sciensano, Juliette Wytsmanstraat 14, Brussels, 1050 Belgium; 2 Interuniversity Institute for Biostatistics and statistical Bioinformatics, Hasselt University, Martelarenlaan 42, 3500 Hasselt, Belgium; 3 Centre for Health Economic Research and Modelling Infectious Diseases (CHERMID), Vaccine & Infectious Disease Institute (WHO Collaborating Centre), University of Antwerp, Universiteitsplein 1, 2610 Wilrijk, Belgium; 4 Epidemiology and social medicine (ESOC), University of Antwerp, Universiteitsplein 1, 2610 Wilrijk, Belgium

## Abstract

**Background:**

Inverse probability weighting (IPW) methods can be used to estimate the total number of cases from the sample collected through sentinel surveillance. Central to these methods are the inverse weights which can be derived in several ways and, in this case, represent the probability that laboratory (lab) sentinel surveillance detects a lab-confirmed case.

**Methods:**

We compare different weights in a simulation study. Weights are obtained from the proportion of participating labs over all labs. We adjust these weights for attractiveness and density of labs over population. The market share of sentinel labs, as estimated by the econometric Huff-model, is also considered. Additionally, we investigate the effect of not recognizing sentinel labs as sentinel labs when they report no cases. We estimate the bias associated with the different weights as the difference between the simulated number of cases and the estimate of this total from the sentinel sample.

As motivating data examples, we apply an extended Huff-model to four pathogens under laboratory sentinel surveillance in Belgium between 2010 and 2015 and discuss the model fit. We estimate the total number of lab-confirmed cases associated with Rotavirus, influenza virus, *Y. enterocolitica* and *Campylobacter spp.*. The extended Huff-model takes the lab-concept, the number of reimbursements and the number of departments, lab-density, regional borders, distance and competition between labs in account.

**Results:**

Estimates obtained with the Huff-model were most accurate in the more complex simulation scenarios as compared to other weights. In the data examples, several significant coefficients are identified, but the fit of the Huff-model to the Belgian sentinel surveillance data leaves much variability in market shares unexplained.

**Conclusion:**

The Huff-model allows for estimation of the spatial and population coverage of sentinel surveillance and through IPW-methods also for the estimation of the total number of cases. The Huff-model‘s gravity function allows us to differentiate inside an area while estimating from the full dataset. Our data examples show that additional data on the participation to surveillance and practices of labs is necessary for a more accurate estimation.

## Background

Sentinel surveillance is defined as; ‘Surveillance based on selected population samples chosen to represent the relevant experience of particular groups’ [1]. Primary objectives of sentinel surveillance are signaling trends [2] and the early detection of events, such as outbreaks [3–5]. Participants to sentinel surveillance are selected such that representativeness, either by qualitative [6] or quantitative approaches [7], for the aspects under surveillance is reached. Since only a selection of health care providers participate to sentinel surveillance, the total number of cases cannot be obtained directly from the output. We can however estimate this total using inverse probability weighting with the probability of detection by sentinel surveillance as inverse weight. Ideally, the proportion of the population that is surveyed by the sentinel network is a known quantity e.g. in primary-care sentinel networks a fixed patient list might exist [8, 9]. These proportions can then be used to obtain weights directly. For other surveillance networks however, weights might not be available directly. The desired proportions [10, 11] will ideally have been established during the design of a sentinel network, but since the actual network often is a network of convenience, driven also by resource restraints and the need for voluntary participation, the actual proportion of the population under surveillance often is unknown [4]. In addition, it is not necessary to know the size of the proportion to fulfill the primary objectives of sentinel surveillance, as long as the proportion remains constant. How best to estimate the probability of detection by the sentinel surveillance therefore has mostly been a topic of discussion among those interested in incidence-estimation from sentinel data [12, 13].

The probability of detection, in this paper represented by inverse weights, can be estimated in different ways, each associated with different assumptions for unbiased estimation. Weights can be estimated from the design of the surveillance. If we make the assumption that the average participant will detect the same number of cases as the average non-participant located in the same area, then the proportion of participants out of all health care providers can be used as an inverse design-based weight. It is possible however that participating to sentinel surveillance is determined by participants’ characteristics. If these characteristics are also related to the detected number of cases, then the earlier stated assumption is violated. Since some of these characteristics might be spatially clustered, we can minimize their confounding effects on the estimation by estimating the total number of cases for smaller, more homogeneous (with respect to these characteristics) areas. Likewise, we might opt to estimate over smaller, more homogenous time periods. Such solutions however are limited; smaller areas need to sum to the total area, areas without participant will be unrepresented and smaller areas will increase variability in estimates. In addition, it seems likely that some confounding characteristics will not be spatially or temporally clustered. To correct for such characteristics, we need to identify and quantify them and allow them into the weight estimation procedure [14]. For example, by adjusting the proportion of participants for the attractiveness of the participant (e.g. the size or expertise of the participant), the attractiveness is now no longer necessarily independent of participating to sentinel surveillance and of the number of detected cases. Souty et al. recently published an overview of such (adjusted) design-based weights that can be used for incidence estimation from sentinel data [15].

As an alternative to the design-based weights we suggest the use of market share as estimated by the Huff-model to derive weights [16]. A market share is defined as the percentage of health care an institution is providing for a certain area. For a lab sentinel surveillance network, this would represent the proportion of lab tests performed on samples from a certain area by a lab out of all lab tests on samples from that area. For a general practitioners network this would represent the proportion of consultations performed in a certain area by a general practitioner out of all consultation performed in that area. As in retail, the distance or accessibility, a convenient location, is an important consideration for patients looking for healthcare [17]. The Huff-model contains a distance decay function to model this effect on market shares. Generally, a provider will have less market share in an area further away [16]. The Huff-model further takes competition between providers into account when estimating detection probabilities and allows for aspatial attractiveness (e.g. the expertise or size of the lab), which influences the market share irrespective of the distance.

The Huff-model has previously been used to estimate catchment areas for and access to health care institutions and to predict how a new institution might affect the workload of existing institutions [18–21]. The Huff-model has also been used to decide on the optimal number and location of participants to sentinel surveillance [10].

### Objectives of this study

We will focus on laboratory sentinel surveillance. Our main objective is the estimation of the total number of newly lab-confirmed cases during a year in the area under investigation, Belgium, from a sample of lab-confirmed cases detected by the sentinel surveillance using IPW-methods. We compare how well design-based (adjusted) weights and the weights estimated by the Huff-model can estimate the total number of cases in a simulation study. We then apply the best performing method to data from the Belgian laboratory sentinel surveillance. Labs that are not reporting cases are considered non-participants to sentinel surveillance by default. We investigate the effect of this assumption, by including the true participation status (in the simulation study) or an estimate for participation (in the data examples) in the analysis.

## Methods

### Methods for estimating the total number of incident lab-confirmed cases

Inverse probability weighting methods use weights to compute linearly weighted estimates of totals. The weights (*w*_*a*_) are an estimate of the inverse of the probability of detection by the sentinel surveillance. To estimate the total number of cases a Horvitz-Thompson-type estimator is calculated as [22];

$$ {\hat{N}}_{national}=\sum \limits_{a\in national}{w}_a\ast Number\ of\ {detected\ cases}_a. $$

The estimated total ${\hat{N}}_a$ for an area (*a*) is obtained by multiplying the number of cases detected by the sentinel surveillance by the inverse probability of detection. The sum of the areas needs to equal the total area of interest. The total area of interest of this study is Belgium (*national*). We use the existing administrative divisions to divide this area in smaller sub-areas: NIS5 < NIS2 < Provinces<Regions<National (from smallest to largest). We estimate the total number of cases by province using design-based weights.

#### Design-based weights

An unadjusted estimate $\left(\hat{N}\right)$ is obtained by using the proportion of reporting sentinel labs over all labs as weight.

$$ {\displaystyle \begin{array}{c}\frac{1}{w_a}=\frac{Sentinel\ {labs}_a}{all\ {labs}_a} = probability\ of\ detection\  by\  sentinel\ surveillance,\\ {}\hat{N}=\sum \limits_{a\in national} Number\ of\ detected\ {cases}_a\ast {w}_a\end{array}} $$

By adjusting the weight for auxiliary information ***x***, we account for the effect of this auxiliary information on the probability of detection by sentinel surveillance. We account for lab-density (= $\frac{\# labs}{\# population}$ at the NIS2-area where the lab is located) and lab-attractiveness (a quantity simulated for each lab) using a direct approach $\left({\hat{N}}_{dens}\  and\ {\hat{N}}_{attr}\right)$ and a calibrated approach $\left({\hat{N}}_{attr. cal}\  and\ {\hat{N}}_{dens. cal}\right)$ [14].

We calculate lab-density for the NIS2-areas (*a*_1_), we use it to adjust the provinces’ weights (*a*_2_). One province contains several NIS2-areas. When we sum over all NIS2-areas, all values of the corresponding provinces will be included.

$$ {\displaystyle \begin{array}{c}1/{w}_{a_2}=\frac{Lab- density\left( Sentinel\ {labs}_{a_2}\right)}{Lab- density\left( all\ {labs}_{a_2}\right)},\\ {}{\hat{N}}_{dens}=\sum \limits_{a_1\in national}\frac{m_{a_1}}{m_{a_2}}\ast Number\ of\ detected\ {cases}_{a_1}\ast {w}_{a_2}.\end{array}} $$

with ${m}_a=\frac{\#{labs}_a}{\#{population}_a}$ calculated on the NIS2 (*a*_1_) and province-level (*a*_2._)

${\hat{N}}_{attr}$ is calculated as all attractiveness over the attractiveness of the sentinel labs:

$$ {\displaystyle \begin{array}{c}1/{w}_{a_2}=\frac{Attractiveness{\left( Sentinel\ labs\right)}_{a_2}}{Attractiveness{\left( all\  labs\right)}_{a_2}},\\ {}{\hat{N}}_{attr}=\sum \limits_{a_2\in national} Number\ of\ detected\ {cases}_{a_2}\ast {w}_{a_2}.\end{array}} $$

Calibrated weights are restrained by calibration equations, which guarantee that the weighted sums of the auxiliary variables sum to their observed totals while keeping the new weights as close as possible, as measured by a distance function, to the initial, uncorrected weights. We opt for the linear distance function. Details on the calibrated approach can be found in appendix.

### Huff-model

The Huff-model is a spatial interaction model for retailing and services and belongs to the family of probabilistic market area models [16].

$$ {P}_{aj}=\frac{A_j^{\alpha }{S}_{aj}^{-\beta }}{\sum \limits_{j=1}^n{A}_j^{\alpha }{S}_{aj}^{-\beta }} $$

The Huff-model calculates the ‘market share’ (*P*_*aj*_) of lab *j* for area *a* based on a spatial component *S* (a direct path between the centroid of *a* and address of *j* in km) and an aspatial component, attractiveness *A* (a simulated quantity for lab *j*). Areas are NIS5-areas (municipalities). Since the model is nonlinear (exponential weighting), parameters *α* and *β* are estimated by ordinary least squares regression after applying multiple step log-centering transformation to the variables [23]. Once *α* and *β* are estimated from the sentinel data set, the market share of every lab for every NIS5-area (*a*_3_) and subsequently the market share of sentinel labs for area *a* $\left({p}_{a_3. sentinel}\right)$ can be estimated.

$$ {\displaystyle \begin{array}{c}\frac{1}{w_{a_3}}={p}_{a_3. sentinel}= probability\ of\ detection\  by\  sentinel\ surveillance,\\ {}{\hat{N}}_{Huff}=\sum \limits_{a_3\in national} Number\ of\ detected\ {cases}_{a_3}\ast {w}_{a_3}\end{array}} $$

In contrast to the other weights, the weight associated with the Huff-model allow for incidences and catchment populations to be calculated. In the Huff-model each sentinel lab potentially (depending on the distance decay function) contributes to the weights in all areas. With the other methods, a sentinel lab only contributes to the weight in the area in which it is located. In addition, a detected case contributes to the total in the area from which it originated with the Huff-model, while, with the other methods, cases contribute to the area in which the lab is located. Incidence estimates are obtained by dividing the estimated number of lab-confirmed cases by the total population/100 000 at the NIS5-area. The catchment population is the result of summing the product of the market share of a lab in a community and community population over the communities.

An overview of the different methods is provided (Table 1).

**Table 1:** Design-based calibrated estimators and Huff-model based-estimators

Input	Auxiliary info	Level of aggregation	Name
Number of reported cases by lab and auxiliary info on laboratories	None	Province (auxiliary info on NIS2-level (arrondissement))	*estHT.prov*
	Attractiveness data by lab		*estHT.prov.attr*
	Lab-density (NIS2)		*estHT.prov.dens*
	Attractiveness and density data		*estHT.prov.densattr*
Number of reported cases by NIS5-code and by reporting lab	Attractiveness data and distance data	NIS5 (municipalities)	*estHuff.NIS5*

### Extending the Huff-model

Because we assume that not only the distance determines the spatial relation between a lab and a NIS5-area, but also if and which regional borders have to be crossed and the clustering of labs, we extended the spatial component before we apply the model to the Belgian laboratory sentinel surveillance network. The spatial component is calculated as follows:

$$ {S}_{aj}=\left({D}_{aj}\ast {Rad}_j\ast {D_{aj}}^{Rad_j}\ast {RB}_{aj}\right). $$

The areas *a* represent communities as defined by NIS5-codes (*N* = 589). ***D***_***aj***_ represents the distance (in kilometers, most direct path) between the location of the lab and the centroid of the NIS5-area (since more detailed addresses are not available for the cases). While the Huff-model takes competition into account when estimating market shares by dividing an area’s market over all labs active in the area, we also allow for dependence between the distance function and the number of laboratories in a 20 km radius ***Rad***_***j***_. We introduce a categorical variable to capture the effect of the regional borders, which mostly coincides with the language borders; Regional Border (***RB***_***aj***_). The three factors in the ***RB***_***aj***_**-**variable are the following:

$$ \left\{\begin{array}{c} Same= the\ region\ of\ the\ cases\ equals\ the\ region\ of\ the\  lab\\ {} Brussels= the\ region\ of\ the\ patient\ differs\ from\ the\ region\ of\ the\  lab, which\ is\ Brussels\\ {} All\  else= all\  other\ configurations\ \left(e.g. case\ located\ in\ Walloon\ region, lab\  located\ in\ the\ Flemish\ region\right)\end{array}\right. $$

Because we have no single measure for the attractiveness of a lab (*A*_*j*_), we included the following variables to calculate the aspatial attractiveness component;

$$ {A}_j={RS}_j\ast {LC}_j\ast {LD}_j\ast {b}_j. $$

The number of reimbursements per lab (***RS***_***j***_) is a continuous variable representing the number of tests for which lab *j* is reimbursed. Depending on the pathogen under investigation this can be a number specific to that pathogen or specific to a group of pathogens. A lab-concept variable (***LC***_***j***_) is introduced to separate ‘hospital-associated’ laboratories from peripheral labs. The factor is coded ‘H’ for ‘hospital-associated’-laboratories and ‘P’ for peripheral labs. The number of different departments of a lab is coded ***LD***_***j***_. A department refers to a different location at which lab-tests are also performed. A normally distributed random effect for the laboratories (***b***_***j***_) is also included. Data from the sentinel labs are used for the model fit. An intercept is included in the model. Model coefficients for which the 95% confidence interval contains zero are set to zero. Details on the extended Huff-model and the coefficient estimation are provided in Appendix.

A 95% bootstrap-based confidence interval is obtained by resampling the records used for the coefficient and market share estimation. A total of 1000 bootstrap samples are used for the computation of each confidence interval.

To limit the variability associated with small probabilities, we do not estimate the total number of cases in areas with a weight higher than 10 (or sentinel coverage lower than 10%). This is done for the design-based estimators and the Huff-model.

### Sentinel-status and zero-reporting

To study the influence of knowledge on the participation status of labs, in contrast to having to assume no participation when no cases are reported, we calculate estimates using prior knowledge on the participation status within the simulation study and estimate the participation of labs in our data example. An additional ‘.S’ (e.g. *estHuff*. *NIS*5. *S*) is added to the estimator to distinguish it from the estimators for which participation is inferred from reporting (e.g. *estHuff*. *NIS*5) in the simulation study. We estimate participation as a discrete quantity between 0 and 1 in the data example. Whenever a lab is reporting cases associated to a certain pathogen, its participation status is 1 for that pathogen. If a lab reports no cases for a certain pathogen, its participation status is estimated from that labs’ reporting of other pathogens. We calculate this quantity (*Psentinellab*) as how many of the twelve most frequently reported pathogens, including viruses (e.g. *influenza*, *rotavirus*), parasites (e.g. *Cryptosporidium spp.*, *Giardia spp.*) and bacteria (e.g. *Yersinia enterocolitica*, *Streptococcus pyogenes*), are reported by that lab.

$$ Psen\mathrm{t} inellab=\left\{\begin{array}{c}1\ \left( if\ cases\ for\ the\ pathogen\ of\ interest\ were\ reported\right)\\ {}\ \frac{\mathrm{x}\ }{12}\ \Big( if\  no\  cases\ for\ the\ pathogen\ of\ interest\ were\ reported\end{array}\right. $$

With x being the number of pathogens (out of the 12 most frequently reported pathogens) for which cases were reported by the lab for which we estimate *Psentinellab*.

If a lab is reporting cases associated with all twelve frequently reported pathogens, it will be given a participation status of 1. If it is reporting cases associated with six of these pathogens, it will be given participation status of 0.5, etc. A participation status of 0.5 will mean that only 50% of the market share of that lab is added to the total sentinel market share. In the data example estimators obtained with an estimated participation-status are marked by adding *Psentinel* to the name of the estimator.

### The simulation study

#### Varying scenarios: detecting cases

We generate a variable number of cases (range: 100–1000) who are detected by 161 labs of which a varying number participates in sentinel surveillance (range: 20–100). The total number of labs as well as the location of the labs in the simulation study corresponds to that of the labs accredited for microbiology in Belgium. Cases are given a NIS5-location. In the simulation study, the background incidence over the different areas is constant. We obtain this constant background incidence by assigning NIS5-areas according to the population size at that NIS5-area. Cases are detected by labs under four different detection-scenarios. In general, we vary the probability with which a case is detected by a certain lab. In the first scenario, all probabilities are equal; cases are detected by a lab irrespective of the distance between the lab and the case or the lab’s characteristics. In the second and third scenario it is either the distance or the attractiveness that determines the probability by which a specific lab will detect a case. In the fourth scenario, the probability is determined by a combination of both; we multiply the attractiveness by the distance decay. The exponential distance decay function is: (*most direct path in km*) ^ (−0.5). We add 0.1 km to the distances equal to 0 (lab at the centroid of the NIS5-area). The distance decay function is chosen such that a lab located 10 times further is 10 times less likely to detect a case. The attractiveness is sampled from a multinomial: *c*(1, 100, 1000, 5000, 10 000), *p*(2/3, 8/30, 1/30, 1/30). The most attractive lab therefore is 10 000 times more attractive than the least attractive lab. Because of the steep distance decay function and the high differences in attractiveness, most cases are detected either by the closest lab or by one of the most attractive labs in scenario 4. Each scenario was run 1000 times for each number of varying cases or labs (Table 2).

**Table 2:** Overview of the simulation scenarios

Detection scenario	Participating labs	Number of cases	Probability of detection	
			Attractiveness of the labs:	Spatial heterogeneity
Random	Labs are randomly selected. The number of labs varies from 20 to 100 (20, 40, 60, 80, 100)	100–1000 (100, 150, 200, 300, 400, 500, 600, 700, 800, 900, 1000)	Uniform	Uniform
Distance			Uniform	Distance decay
Attractiveness			Multinomial	Uniform
Attractiveness and distance			Multinomial	Distance decay

#### Performance of the methods

We calculate the RMSE as;

$$ RMSE=\sqrt{\frac{\sum \limits_{i\to n}{\left(N-{\hat{N}}_i\right)}^2}{n}} $$

*N* represents the total number of lab-confirmed cases, $\hat{N}$ represents an estimate of the total number of lab-confirmed cases, *n* represents the number of samples. The RMSE is presented over the number of cases and labs.

### The data of the Belgian sentinel laboratory surveillance

Data are provided by the Belgian laboratory sentinel surveillance network. This network was previously described in Muyldermans et al. [24]. The network relies on voluntary participating labs that submit data on a set of around 35 pathogens. The list of pathogens under surveillance is constituted during a yearly meeting. The reported data consist of patient demographic data; postal code, date of birth and gender and data on the diagnosis; date of diagnosis, subspecies and type of test. We limit the dataset to data from 2010 to 2015 and to four pathogens; *Yersinia enterocolitica*, *influenza, campylobacter spp* and *Rotavirus*. We chose four commonly reported pathogens that were under surveillance before, during and after the period 2010 to 2015. An additional motivation for the choice of pathogens was that these represent the different categories of the nomenclature numbers; a pathogen-specific, a group-specific and a non-specific number.

Data on reimbursed microbiology tests were obtained from the Belgian National Institute for Health and Disability Insurance (INAMI-RIZIV) for the period 2010–2015. The data consist of the number of reimbursed tests by nomenclature number, year and lab. A specific nomenclature number is available for Rotavirus antigen-test or culture*.* A number associated with a test for a group of enteric pathogens (< 6) is available for *Yersinia enterocolitica* and *Campylobacter spp.*. No specific nomenclature is available for influenza*.* When no specific reimbursement data is available the total number of reimbursements is used.

Some labs have reference activities with regards to specific pathogens. These reference-labs are identified and removed from the dataset for estimation of the total number of lab-confirmed cases. Once the estimation process is completed, their data are added to the estimated total. We exclude the data from reference-labs from the estimation process because these labs have several unique characteristics and are not representative for the other labs. For example, we assume their distance decay function will be less steep as compared to the average distance decay. They do however detect cases and it is essential to allow them to contribute to the total number of lab-confirmed cases.

Data on the location of microbiological labs is also obtained from the INAMI-RIZIV. Data on the Belgian demographics for the period 2010–2015 is obtained from the general directorate statistics Belgium.

## Results & interpretation

**Fig. 1:**
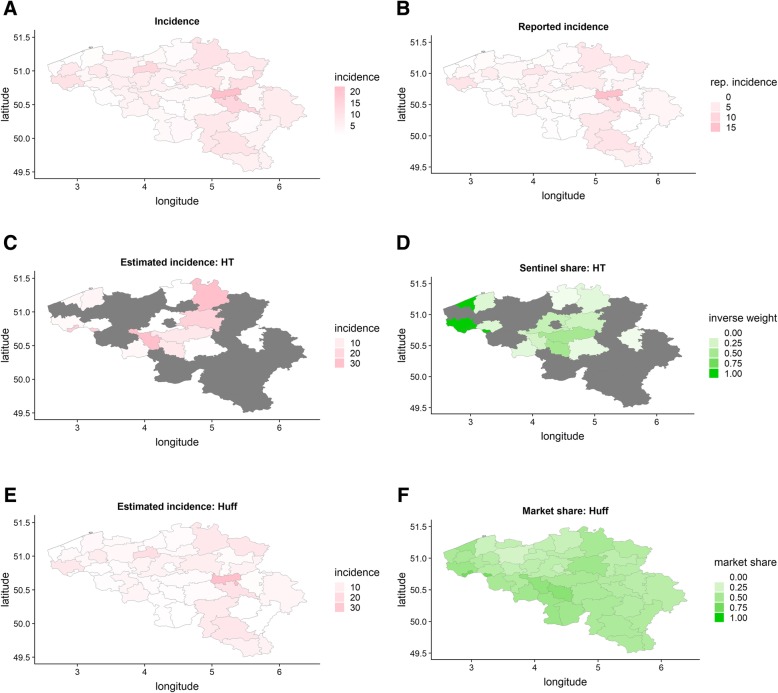
The Belgian NIS2-areas filled in for: the simulated incidence (**a**), the reported incidence (**b**), the market share of the sentinel surveillance (**f**), the proportion of sentinel labs over all labs (**d**), incidences obtained with the Huff-model (**e**) and obtained with unadjusted design based weights (**c**)

We provide an illustration of the output of a single simulation run from scenario 4 (both spatial and reimbursement heterogeneity, 80 sentinel labs, 500 cases) in Fig. 1. For this illustration we estimate NIS2-area incidences both with the unadjusted design-based weight and with the Huff-model based weights. As stated in the methods-section, the incidence with the design-based weight is to be interpreted as the number of cases detected by sentinel labs in a certain area over the total number of persons in that area. For multiple NIS2-areas a design-based weight could not be obtained since no sentinel labs were active in those areas. They are left gray.

### Results of the simulation

A discussion of the performance of the design-based weights is included in Appendix.

#### Varying the number of cases

**Fig. 2:**
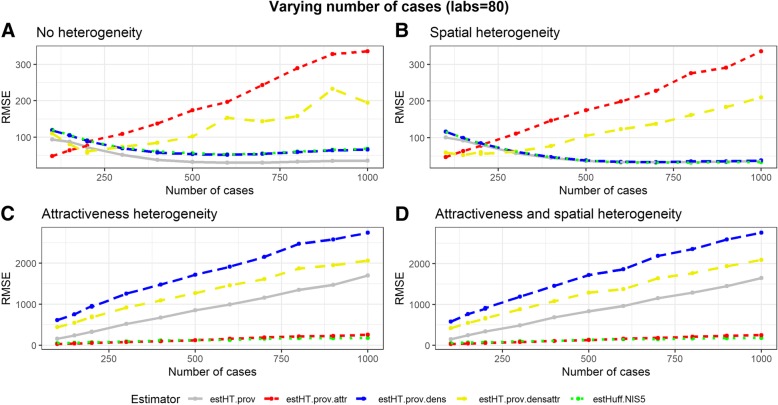
Simulation results for the scenarios: 'No heterogeneity' (**a**), 'Spatial heterogeneity' (**b**), 'Attractiveness heterogeneity' (**c**), 'Attractiveness and spatialheterogeneity' (**d**), over a varying number of cases

Under a varying number of cases the results obtained by the weight estimated by the Huff-model have a RMSE lower than or equal to results obtained using other weights except for the scenario in which all labs have an equal probability of detecting a case (the scenario without heterogeneity). In this scenario the Huff-model is outperformed by the unadjusted design-based weight (Fig. 2).

#### Varying the number of labs

**Fig. 3:**
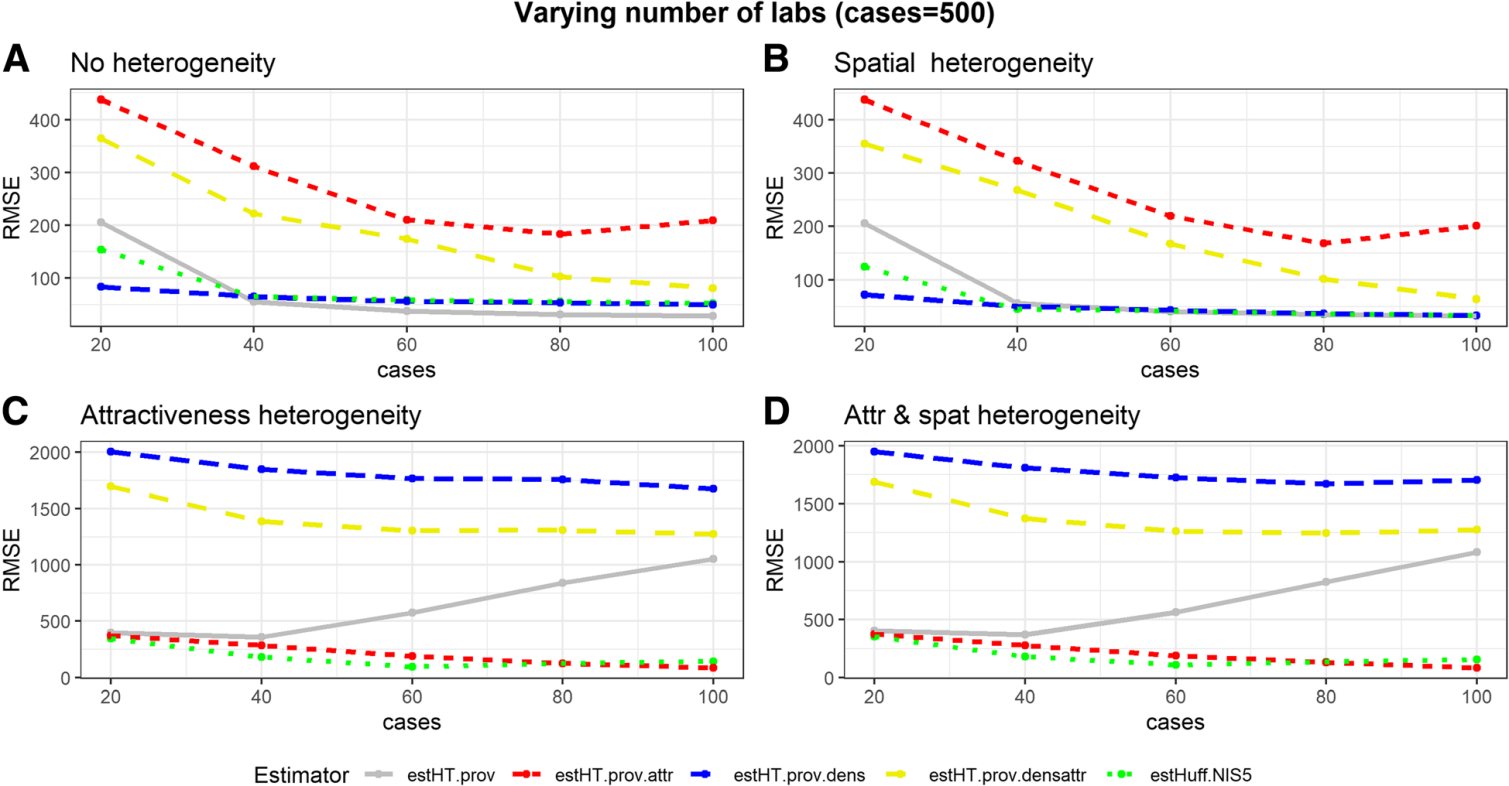
Simulation results for the scenarios: 'No heterogeneity' (**a**), 'Spatial heterogeneity' (**b**), 'Attractiveness heterogeneity' (**c**), 'Attractiveness and spatial heterogeneity' (**d**), over a varying number of labs

Overall the Huff-model performs equally well to or better than the design-based estimators under a varying number of labs. There is one exception; the spatial scenario with only 20 reporting labs. The higher RMSE in this scenario is due to our choice to eliminate areas with a weight higher than 10. Whenever only 20 labs participate to sentinel surveillance, the mean weight is 8.05 (=161/20). In the random scenario, in which all labs have the same market share, this weight is small enough not to eliminate too many areas. In a scenario with heterogeneity, areas will be removed due to having too high a weight (Fig. 3).

#### Including the sentinel-status

**Fig. 4:**
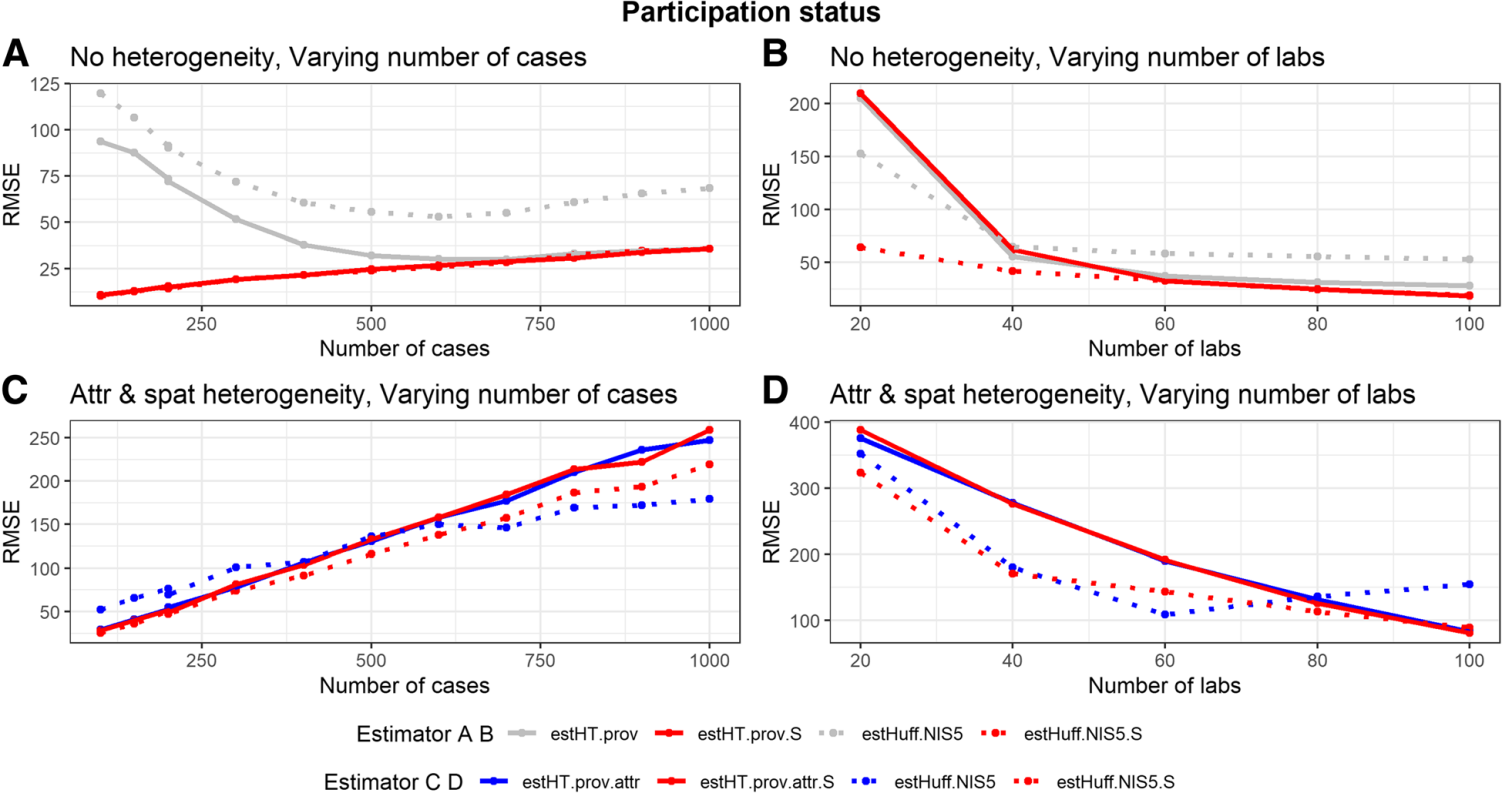
Influence of the participation status in scenarios with (**c** & **d**) and without (**a** & **b**) heterogeneity

After including the sentinel-status non-reporting sentinel labs are recognized as sentinel labs. The Huff-model outperforms the unadjusted weight in scenarios without heterogeneity. This is especially true in scenarios with few cases or few sentinel labs. While the effect is smaller, it is also present in scenarios with spatial and attractiveness heterogeneity (Fig. 4). A more detailed description of the effect of including the sentinel-status is provided in Appendix.

### Incidence estimation from the Belgian laboratory sentinel surveillance dataset

**Fig. 5:**
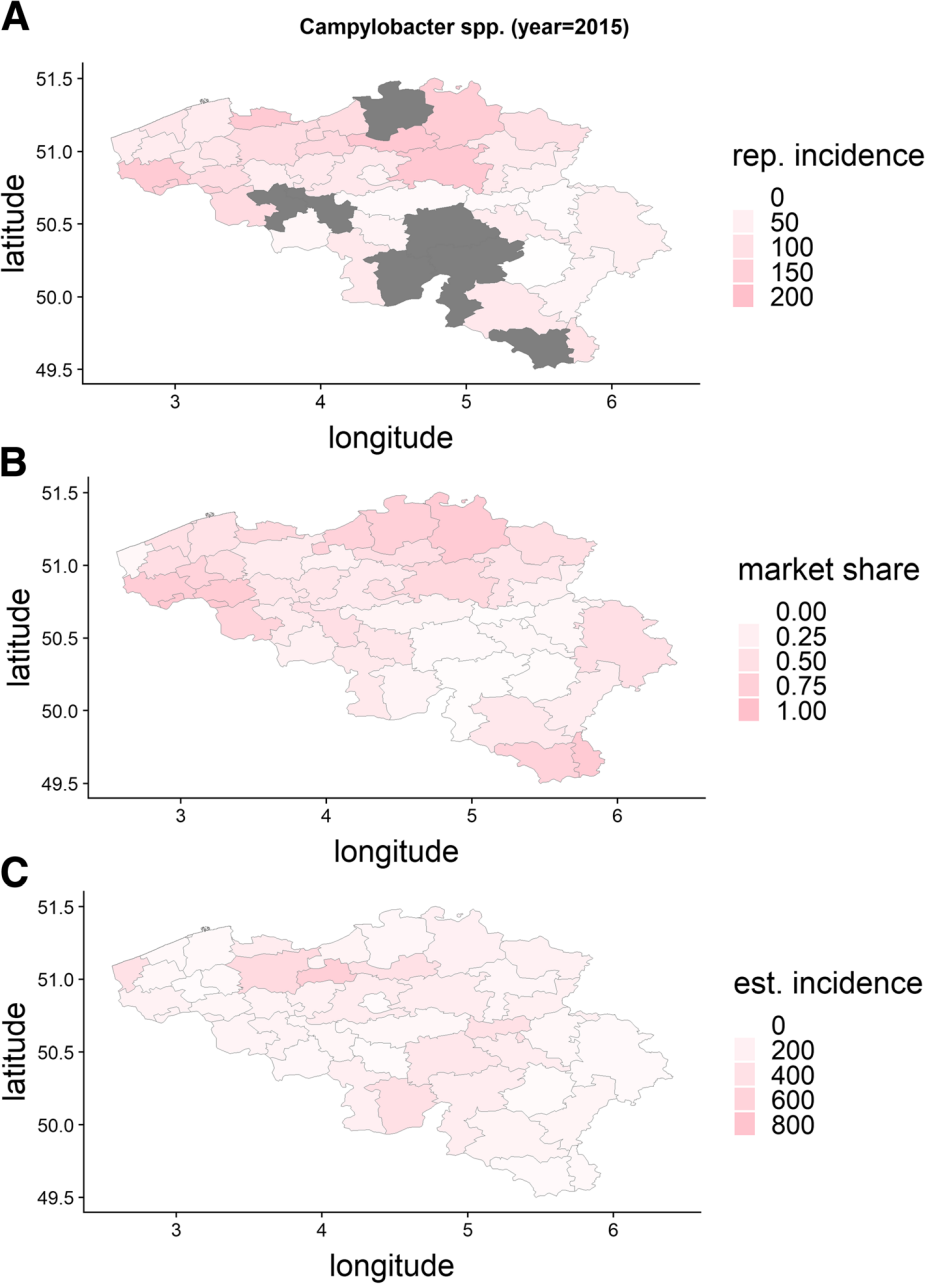
Reported (**a**) and estimated (**c**) incidence (/100 000 persons) and market share (**b**) per NIS2-area for *Campylobacter spp.* 2015, Belgian laboratory sentinel surveillance

We illustrate the model by presenting the reported and estimated incidence and estimated market share for *Campylobacter spp.* for 2015 (Fig. 5).

#### Model coefficients

For all four pathogens, the market share of a lab decreases as the distance from a lab to an area increases. This effect interacts with the number of labs in close proximity. Labs have a further reach if a higher number of other labs are present in a 20 km radius. The market share is larger when a lab requested more reimbursements. This effect is largest for *campylobacter spp.*. Whenever the lab is located in Brussels its market share in the other regions is smaller. These effects are significant for all pathogens. A higher number of departments also increases the market share for labs for *campylobacter spp.,* but not for the other pathogens. The lab-concept, peripheral labs vs. hospital associated labs, has no significant effect for any of the pathogens. The precision of the lab random variable is low for influenza (Table 3).

**Table 3:** Model coefficients for the Extended Huff model, 2015, Belgian laboratory sentinel surveillance (*se* standard error, *P* peripheral, *H* Hospital, *represents an interaction term)

Fixed effects: mean (se)	*Campylobacter spp.*	Rotavirus	Influenza	*Y. enterocolitica*
Intercept	0.16 (0.06)	0.31 (0.09)	0.43 (0.19)	0.31 (0.10)
Distance	− 2.52 (0.03)	−1.96 (0.02)	−2.64 (0.03)	−0.89 (0.02)
Reimbursements	0.42 (0.06)	0.31 (0.10)	0.18 (0.08)	0.19 (0.09)
Lab-concept P (vs H)	−0.14 (0.14)	−0.21 (0.25)	−1.09 (0.86)	− 0.33 (0.31)
Labs in Radius	−0.10 (0.05)	− 0.16 (0.08)	−0.11 (0.13)	− 0.02 (0.07)
Distance * Labs in Radius	0.16 (0.04)	0.425 (0.03)	0.40 (0.05)	0.24 (0.03)
Number of departments	1.74 (0.27)	0.49 (0.44)	1.02 (0.92)	−0.18 (0.24)
Borders: Brussels (vs ‘all else’)	−1.05 (0.20)	−1.95 (0.28)	−4.94 (0.39)	−2.18 (0.14)
Borders: Same (vs ‘all else’)	−0.05 (0.04)	−0.15 (0.03)	− 0.08 (0.04)	−0.05 (0.02)
Random effects: mean (se) of the precision	*Campylobacter spp.*	*Rotavirus*	*Influenza*	*Y. enterocolitica*
Lab-effect	11.26 (3.50)	4.12 (1.30)	0.67 (0.17)	2.7 (0.61)

#### Model fit

**Fig. 6:**
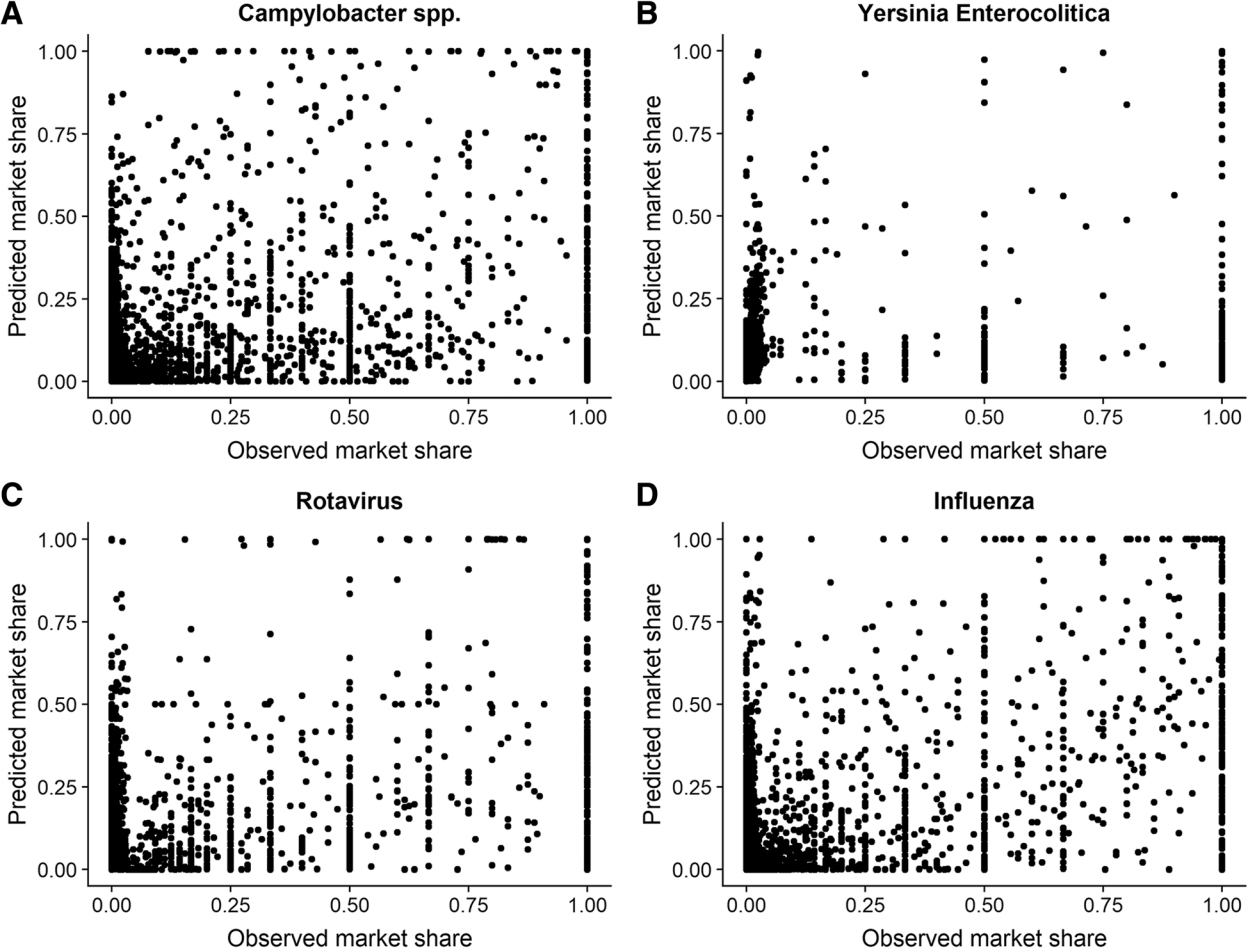
Predicted (y-axis) vs observed (x axis) market shares for the four pathogens ((**a**) Campylobacter spp, (**b**) Yersinia enterocolitica (**c**) Rotavirus, (**d**) Influenza), 2015, Belgian laboratory sentinel surveillance

While the extended Huff model has multiple significant coefficients, the model fit is limited. We illustrate this by plotting the predicted market share to the observed market share for the sentinel surveillance dataset from 2015 for the four pathogens (Fig. 6).

#### Coverage of the sentinel surveillance and incidence estimates

Once all market shares are estimated, which labs participate to the sentinel surveillance will determine the coverage of the sentinel surveillance network. Since we determine participation in two different ways, we present two different coverage estimates.

#### Reporting 0 cases = Not participating

Sentinel labs have a higher market share than the non-sentinel labs. In addition, sentinel labs are located more frequently in the more densely populated north of Belgium. The difference between population coverage and the proportion of sentinel labs over all labs varies from 7% (*Campylobacter spp.*) to 11% (*Y. enterocolitica*) in 2015. The difference between the population coverage and the mean of the market shares, spatial coverage, ranges from 3% for *Campylobacter spp.* to 9% for *Y. enterocolitica* in 2015 (Table 4).

**Table 4:** Coverage estimates and the total number of cases (95% bootstrap-based confidence intervals) for the four pathogens. The presented coverage-estimates are the proportion of reporting laboratories, the catchment population and the area covered by sentinel surveillance (average of the market shares over all NIS5-areas). Participation is set equal to reporting, Belgian laboratory sentinel surveillance

Germ	Reporting 0 cases = Not participating		
	$\frac{Sentinel\ labs}{All\ labs}$	$\frac{Covered\ pop}{Total\ pop}$	$\frac{Covered\ area}{Total\ area}$
Campylobacter spp.	0.45	0.52	0.49
Influenza	0.35	0.43	0.40
Rotavirus	0.43	0.52	0.51
Y. enterocolitica	0.33	0.44	0.35
	Reported Number of cases	Estimated number of cases (95% CI)	
Campylobacter spp.	8813	14918 (11404–15446)	
Influenza	7382	12059 (9454–13827)	
Rotavirus	2750	4325 (3455–4657)	
Y. enterocolitica	370	834 (740–1049)	

**Fig. 7:**
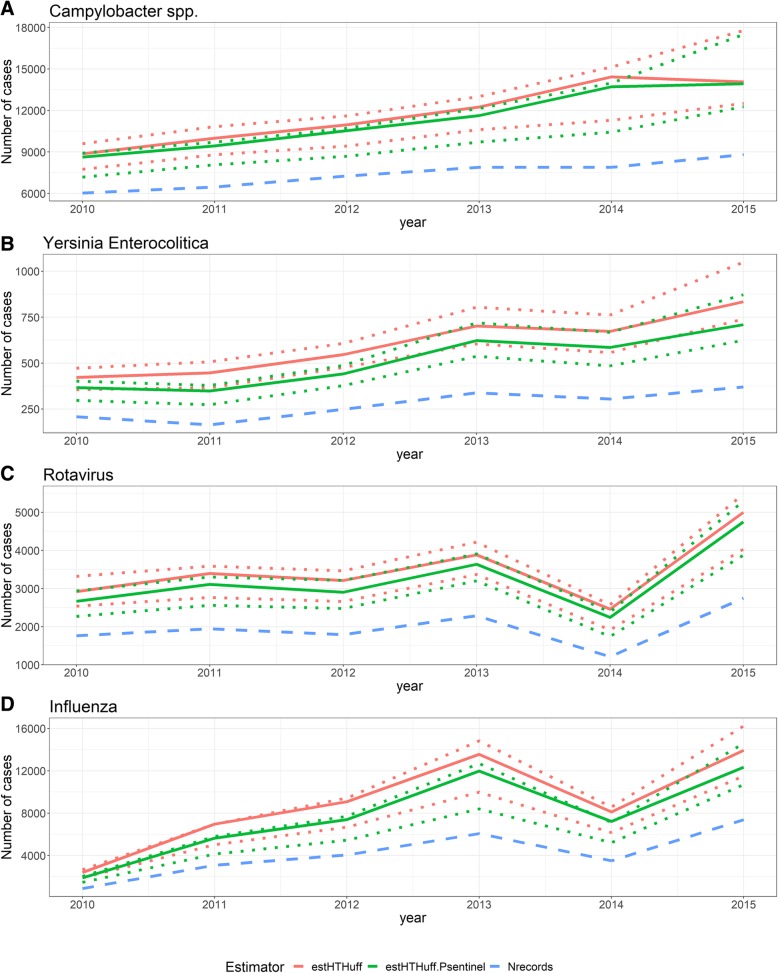
Estimates by the extended Huff-model without (estHTHuff) and with additional estimated participation (estHTHUff.Psentinel) and the number of reported cases (Nrecords) by year (2010–2015) + 95% bootstrap-based confidence intervals (dotted lines) for four pathogens ((**a**) Campylobacter spp, (**b**) Yersinia enterocolitica (**c**) Rotavirus, (**d**) Influenza), Belgian laboratory sentinel surveillance

There is variation in the coverage between pathogens. The population-coverage ranges from 58% for *Campylobacter spp.* in 2010 to 36% for influenza in 2010. There is ‘year to year’-variation for single pathogens. For influenza the estimated spatial coverage of the sentinel surveillance was 33% in 2010 and 41% in 2014. The year to year variation in coverage for specific pathogens is smaller than the variation between pathogens (Fig. 7).

#### Estimated participation

By assigning a participation status to labs that are not reporting cases, the market share of sentinel labs increases. This results in lower estimates for the total number of lab-confirmed cases. Differences vary from 87 cases (*Y. enterocolitica*, 15% of the initial estimate) to 654 cases (influenza, 36% of the initial estimate) (Table 5).

**Table 5:** Coverage estimates and the total number of cases (95% bootstrap-based confidence intervals) for the four pathogens, 2015, Belgium. The presented coverage-estimates are the proportion of laboratories participating in sentinel surveillance, the catchment population and the area covered by sentinel surveillance (average of the market shares over all NIS5-areas). Participation is estimated from reporting the most frequently reported pathogens, Belgian laboratory sentinel surveillance

Germ	Estimated participation (Psentinel)		
	$\frac{\ Sentinel\ labs}{All\ labs}$	$\frac{Covered\ pop}{Total\ pop}$	$\frac{Covered\ area}{Total\ area}$
Campylobacter spp.	0.45	0.54	0.51
Influenza	0.35	0.51	0.48
Rotavirus	0.43	0.54	0.53
Y. enterocolitica	0.33	0.52	0.45
	Reported Number of cases	Estimated number of cases (95% CI)	
Campylobacter spp.	8813	14438 (11110–14942)	
Influenza	7382	11405 (9338–13010)	
Rotavirus	2750	4203 (3395–4679)	
Y. enterocolitica	370	585 (485–667)	

## Discussion

In this study we compare different weight estimation procedures for use in IPW-methods to estimate the total number of lab-confirmed infectious disease cases from sentinel surveillance data with a simulation study. We find that the weight estimated with the Huff-model, the market share, outperforms the use of other weights in most simulation scenarios. In addition, market shares have several desirable characteristics.

- With the Huff-model, the weight is the result of a fitting process. With the other methods we obtain the weight more directly as an (adjusted) proportion. Directly adjusting the weight will result in a large RMSE if what is adjusted for does not determine the number of cases reported by the lab (e.g. adjusting for lab-density in the attractiveness heterogeneity simulation scenarios). The market share won’t be biased in a comparable way from introducing unrelated variables into the Huff-model. In the absence of confounding the coefficients will not be significant and will not be used for further estimation.
- The second important difference is how the distance decay function allows us to differentiate inside an area in a data-driven way. The probability to detect cases decreases as the distance between case location and lab increases. With the other weights, the area that is aggregated over only has one denominator, so all additional spatial information from within that area is lost. Choosing an area to aggregate over is not a straightforward task and will be a compromise between eliminating differences between labs by only aggregating over labs in close proximity and having areas large enough to eliminate unwanted variability. Not only the size, but also the shape of the subareas will define the estimates. Administrative areas are readily available and an interest might exist in these specific areas, but labs might be located close to the borders and therefore influenced more by neighboring areas.
- Finally the interpretation of the obtained estimates is quite different; with the Huff-model we obtained the number of cases per area, with the other methods we obtained the number of cases detected by labs in the area.

### Limitations of the simulation study

The simulation study mirrors some aspects of the Belgian sentinel labs surveillance network. These include the location of the labs, areas of aggregation and associated population sizes which were used to allocate cases to NIS5-areas. We explored this approach previously with the application of capture-recapture methods to surveillance data [25]. Such an approach however has different limitations, most importantly; the results of the simulation study are specific to this setting. In the simulation study several aspects are simplified, for example, we only simulate cases from a constant background incidence. Additionally we also opt for a simple case-definition without differences between cases and the heterogeneity in the detection probability of the labs is limited. With a real dataset, we might expect design-effects such as higher detection probabilities for more severe cases [26].

While adjusting for lab-density did not improve any of our estimates, such a variable could be of interest if it was computed differently. Souty et al. found that including the density of GPs over population improved their IPW-estimate [15]. In our simulation study it was driven by the administrative division in NIS2-areas. Future investigations could focus on the effect of lab-density calculated over custom areas with the labs at their center.

### Limitations of the estimation of the market shares

Market shares are, in contrast to design-based weights, not directly available. We need a model to estimate them. We find that the model fit is limited. This can be caused by both; a lack of power and unmodelled heterogeneity in the detection of cases by labs. To increase power, we could combine several years of observations and include a temporal component into the analysis. In addition, longitudinal analysis, e.g. a time-series analysis would allow for the smoothing of weights, which could be a solution for the high weights encountered in this study [27]. Furthermore, longitudinal models might be used as possible outbreak/early event detection tools. Variation in coefficients (e.g. positivity rate of reimbursed tests) over time has been proposed as an outbreak detection tool [28]. Keeping in mind however that temporal variation might also be the consequence of changes in lab tests or policies [29]. It is likely also possible to combine data from similar pathogens to increase power. In addition to the power necessary to estimate the coefficients of the extended Huff-model, sentinel surveillance should also have enough power, high enough market shares, in the areas of interest to detect events and describe trends. The Huff-model allows us to optimize power by taking lab-profiles into account [30]; market shares can be used to estimate both spatial and population coverage associated with a specific lab. We find that; the Belgian laboratory sentinel surveillance is a network of convenience, with a high geographical overlap in the north of Belgian and low market shares central and south. This unequal spatial coverage in combination with an exponential distance decay function will lead to very small market shares in some areas and consequently to unstable estimates and a reduction of the effective sample size [31]. An arbitrary cut-off or piecewise distance decay function can be used to avoid a laboratory’s small market shares in distant areas, but might lead to artificial observations. E.g. our choice to ignore weights larger than 10 reduces the instability of the IPW-estimator, but also leads to a considerable RMSE for the Huff-model when there are few sentinel labs in a simulated scenario with spatial heterogeneity. Other techniques to improve the efficiency of IPW have been explored in epidemiology, notably weight stabilization and augmented IPW [31].

Among the significant coefficients we did find, we found a significant and positive relation between the number of reimbursements and the market share in all data examples. We had however anticipated a higher effect size. Our results are in accordance with previous research that established a large variation in lab use by physicians [32]. We are likely missing important variables which help determine labs’ market shares. Several of these will be hard to capture as they can be pathogen and time-specific. For example; an informal referral system might exist among the labs. Referring samples is straightforward as they are easy to transport. What constitutes the attractiveness of a lab for cases will likely be different from what constitutes attractiveness for other labs. Another possible cause of heterogeneity in observed market shares are local outbreaks that are unaccompanied by an equal increase in testing behavior and that are not equally detected by all labs active in that area. Consider, for example, an outbreak in a hospital linked to one specific lab. An increase in tests due to a local screening program or change in attention/attitude that is not shared by all labs active in an area, can also cause a limited model fit. State specific health policies are a known cause of spatial heterogeneous reporting in sentinel surveillance and could further explain our limited model fit [11]. Health policies relevant to laboratory testing however are tied to the federal level and therefore common over all of Belgium. Public health policy did establish reference centers for laboratory tests. We excluded these labs from the estimation process because of their unique position.

In econometric studies ‘past performance’ is sometimes included as laboratory/store-specific variable [33]. With such a variable it is not necessary to identify all elements contributing to attractiveness. The significance of the random effect in our extended Huff-model points to the usefulness of such a parameter. Unfortunately such a variable would not be available for many labs as they have never participated in sentinel surveillance and it is not possible to estimate this for non-participating labs. A one-time measure, either through a survey or other data collection method could improve the model fit.

Finally, data quality issues cannot be remedied by our model and will contribute to a poor model fit. We assume reporting consistency on a yearly basis; a lab that is participating to sentinel surveillance should report all detected cases. Incomplete reporting (not reporting all detected cases), low data quality (typos, missing variables such as case location) and variations in the case-confirmation/definition among laboratories (only reporting on cases detected with a certain test or not being able to perform a certain test) will lead to a poor model fit.

### Limitations of the estimation of the incidence

A previous estimate of the representativeness of the Belgian laboratory sentinel surveillance network, obtained through analysis of the proportion of tests reimbursed by labs participating to sentinel surveillance, has found a coverage of around 50% [34]. The central assumption of that study was that sentinel labs report an equal number of cases as non-sentinel labs given correction for the number of reimbursed tests. We opt for a less strict assumption, by also correcting for other variables, such as lab-density, location and regional borders.

The absence of a participation status, not being able to distinguish zero-reporting labs (labs reporting no cases) from non-participating labs, is a hindrance to all methods and explains why the Huff-model has a larger RMSE is some simulation scenarios. This is an important limitation of the Belgian laboratory sentinel surveillance. This is further complicated by small yearly changes to the list of pathogens under surveillance. The assumption that a sentinel lab that did not report any cases did not participate and the assumption that laboratories that are not reporting any cases participate with a probability estimated from the reporting of other, frequently reported, pathogens are both strong. To illustrate this, we present some observations. Eighty labs, out of 161 microbiological labs, have participated to sentinel surveillance in 2015. A total of 35 pathogens were under surveillance in 2015. No lab reported cases for all the pathogens, two labs reported cases for more than 80% of the pathogens, 28 labs reported cases for more than 50% of the pathogens under surveillance in 2015. So even for common pathogens such as Rotavirus or influenza only 88 and 58% of all sentinel labs reported cases. In addition to a participation status to sentinel surveillance, it would be essential to know if a lab has the technical capacity to perform a certain test. Throughout this paper we assumed that all labs could detect all cases.

### Confidence interval and full probability models

We apply a bootstrap algorithm for the estimation of confidence intervals. This is time consuming and analytical methods are available for direct confidence interval estimation even in a small area estimation setting. For example; using the weights obtained from the sampling design, effective sample sizes can be calculated [26]. Other methods are available to obtain a full probability model for small area estimation [35]. The nature of sentinel surveillance data, the quality and methodology of the systems, might however make additional variance inflation components necessary [36]. Additional research into the unmodelled heterogeneity is necessary to come to such a variance inflation component. Future extensions to the Huff-model include informative priors in a full Bayesian framework. Sample weights have already been incorporated in 3-stage Bayesian hierarchical models, including variance estimation [26]. Other methods (optimization algorithms such as harmony search) have also been developed to estimate the coefficients of the Huff-model [10].

## Conclusion

We suggest the use of Huff-model based weights for estimating the coverage of a sentinel surveillance network and the total number of lab-confirmed cases. Epidemiologists can tailor fit the Huff-model to a specific surveillance setting in a data-driven way. We estimate the population-coverage of the Belgian laboratory sentinel surveillance from 36 to 58% for four pathogens under surveillance from 2010 to 2015. Even though the model fit to the observed sentinel market shares is limited, several coefficients are found to be significant. Data on the participation of labs to sentinel surveillance would improve all methods under investigation. Variables on laboratory-practices and capacities are not included in our Huff-model, but could be topics of future research.

## Data Availability

All R-code is made available and included as supporting files. The datasets generated and/or analyzed during the current study are not publicly available due to privacy concerns, but aggregated data is available from the corresponding author on reasonable request.

## Appendix

### Calibrated approach

We first introduce the linear distance function:

$$ D\left({w}_{old\  weight},{w}_{new\  weight}\right)=\sum \limits_{i=1}^n{w}_{new\  weight,i}\ast \frac{1}{2}{\left(\frac{w_{old\  weight,i}}{w_{new\  weight,i}}-1\right)}^2 $$).

We calculate ${\hat{N}}_{dens. cal}$ (calibrated on province totals) as;

$$ {\hat{N}}_{dens. cal}\  per\  province=\sum \limits_{a\in prov}{w}_{new\  weight,a}\ast Number\ {of\ observations}_a $$

With the new weights computed as:

$$ {w}_{new\  weight, arro}={w}_{old\  weight, arro}\ast \left(1+\boldsymbol{x}^{\prime }{\boldsymbol{T}}_{\boldsymbol{prov}}^{-\mathbf{1}}\ \left({\boldsymbol{t}}_{\boldsymbol{x}}-{\hat{\boldsymbol{t}}}_{\boldsymbol{x}\boldsymbol{\pi }}\ \right)\right) $$

Where ***t***_***x***_ represents the total of the auxiliary information on the province level and ${\hat{\boldsymbol{t}}}_{\boldsymbol{x}\boldsymbol{\pi }}$ represents the estimate of this auxiliary information calculated using the old weights.

Assuming that the inverse of ${\boldsymbol{T}}_{\boldsymbol{prov}}=\sum \limits_{prov}{w}_{old\  weight, arro}{\boldsymbol{x}}_{\boldsymbol{arro}}\boldsymbol{x}{\prime}_{\boldsymbol{arro}}$ exists. The old weights *w*_*old weight*_ are calculated as; $1/{w}_{old\  weight, arro}={\pi}_{prov}=\frac{{Reporting\ labs}_{prov}}{all\ {labs}_{prov}}$

***x*** can be a single vector, for a single variable, or a matrix, for multiple variables. We calculate the estimate calibrated on attractiveness and lab-density as;

$$ {\hat{N}}_{densattr. cal}\  per\  province=\sum \limits_{arro\in prov}{\pi}_{prov}^{-1}\left(1+{\left({\boldsymbol{t}}_x-{\hat{\boldsymbol{t}}}_{x\pi}\right)}^{\prime}\bullet {\boldsymbol{T}}_{\boldsymbol{prov}}^{-\mathbf{1}}\bullet {\boldsymbol{x}}^{\prime}\right) Number\ {of\ observations}_{arro} $$

where ***t***_***x***_ is the total of ***x*** in the population, ***t***_*xπ*_ denote the HT-estimator for the ***x***− vector with expression, $$ {\boldsymbol{t}}_{x\pi}=\left[\begin{array}{c}\sum \limits_{arro}{\pi}_{prov}^{-1}\bullet \frac{1}{m_{arro}}\\ {}\sum \limits_{arro}{\pi}_{prov}^{-1}\bullet {\rho}_{arro}\end{array}\right] $$ and $$ {\boldsymbol{T}}_{\boldsymbol{prov}}=\left[\begin{array}{cc}\sum \limits_{arro}{\pi}_{prov}^{-1}\bullet \frac{1}{m_{arro}^2}& \sum \limits_{arro}{\pi}_{prov}^{-1}\bullet \frac{1}{m_a}\bullet {\rho}_{arro}\\ {}\sum \limits_{arro}{\pi}_{prov}^{-1}\bullet \frac{1}{m_{arro}}\bullet {\rho}_a& \sum \limits_{arro}{\pi}_{prov}^{-1}\bullet {\rho_{arro}}^2\end{array}\right] $$, assuming that the inverse of ***T*** exists. *ρ*_*arro*_ represents the attractiveness of participating laboratories on the NIS2-level. *m* represents the lab-density $=\frac{\# labs}{\# population}.$

### Fitting the extended Huff-model

We estimate the coefficients from the reported (sentinel) data.

1. First prepare a file with all possible combinations between NIS5-areas and labs. For these combinations we calculate the distance between the NIS5-area (in kilometer, most direct path ***D***_***aj***_) and the lab and the factor that represents the regional border (***RB***_***aj***_) variable.
2. For every combination we add the lab-specific variables (the number of other labs in a radius of 20 km (***Rad***_***j***_), the number of departments (***LD***_***j***_), the lab-concept (***LC***_***j***_: whether or not it is a hospital-associated lab). Since we do not have area-specific reimbursement numbers per lab, we add the total number of reimbursements per lab (***RS***_***j***_).
3. Finally, we add the number of cases associated with the combination. (The number of cases detected by a certain lab originating from a certain NIS5-area.)
4. From the number of cases, for each NIS5-area we calculate the observed market share:

$$ Observed\ market\ share\ of\ {lab}_j\  in\ {area}_a=\frac{Number\ of\ cases\ detected\  by\ {lab}_j\  in\ {area}_a\ }{Total\ number\ of\ cases\ detected\ in\ {area}_a} $$

5. Now that all variables are available we log-center the variables to be able to fit them with a linear model later on.

$$ Logcentered(x)=\log \left(\frac{x}{geometric\ mean(x)}\right) $$

The geometric mean (average over the NIS5-area) of variable *x* is estimated as:

$$ geometric\ mean(x)= Exp\left(\frac{\sum \limits_n\left(\log \left(x\left[x>0\right]\right)\right)}{n}\right) $$

*n* represents the number of observations (over the NIS5-area) available for variable *x*. (E.g. if there are 80 sentinel labs active in the NIS5-area, *n* = 80).

6. Once all independent and the dependent variable are log-centered, we use a linear mixed model to fit the data.

$$ lc\left({market\ share}_{aj}\right)={\beta}_1 lc\left({\boldsymbol{D}}_{\boldsymbol{aj}}\right)+{\beta}_2 lc\left({\boldsymbol{RB}}_{\boldsymbol{aj}}\right)+{\beta}_3 lc\left({\boldsymbol{RS}}_{\boldsymbol{j}}\right)+{\beta}_4 lc\left({\boldsymbol{LD}}_{\boldsymbol{j}}\right)+{\beta}_5 lc\left(\ {\boldsymbol{Rad}}_{\boldsymbol{j}}\right)+{\beta}_6 lc\left(\ {\boldsymbol{Rad}}_{\boldsymbol{j}}\right)\ast {\beta}_7 lc\left({\boldsymbol{D}}_{\boldsymbol{aj}}\right)+{b}_j+\varepsilon $$

*b*_*j*_ is a random variable, representing a lab-specific effect (1|lab).

### Results and discussion of the performance of the design-based weights

#### Unadjusted weights

An unadjusted weight will only be unbiased if the average sentinel lab reports an equal number of cases as the average non-sentinel lab in the same area and if all areas contain at least one sentinel lab. The first condition is met in a scenario without heterogeneity, but even with 80 sentinel labs and 250 cases the second condition is likely not met if the chosen areas are provinces and the selection of participating labs is random.

#### Adjusting for lab-density and lab-attractiveness (and calibration)

Adjusting a weight will introduce bias if that what is adjusted for is not related to the number of reported cases by lab. For example adjusting for attractiveness in a scenario in which attractiveness does not determine detection, can lead to both an upward or downward bias. In our simulation study most labs have low attractiveness; therefore the bias will likely be upward. Lab-attractiveness is a lab-specific variable, therefore it is area-independent and calibrated estimates will not differ from non-calibrated estimates. The lab-density is area-specific and therefore sensitive to calibration.

Differences in lab-density between provinces are captured by the unadjusted weight as it is computed on the province level. Differences in lab-density between NIS2-areas are taken into account when lab-density adjusted estimates are computed on the province level. The adjusted weights do not outperform the unadjusted weights in scenarios with spatial heterogeneity and will bias the estimate in some scenarios. The reason for this observation is that cases can be detected over area boundaries, while the lab-density variable is area-specific. The lab-density of surrounding areas also determines the number of detected cases in a certain area, but is not taken into account.

Calibration weighting was introduced as a tool for reducing the standard errors of many, if not most, finite-population estimates. In this study a clear example of an additional benefit, how calibration weighting can remove, or at least greatly reduce, the potential for nonresponse bias in the resulting estimates, is found [37]. The direct adjustment of weights for the density of the labs over the population on the arrondissement-level is biased since some arrondissements do not have any labs. A part of the population is thus not taken into account on the arrondissements level, but is on the province level. The arrondissement population totals do not sum up to the province population total. This leads to estimates for province-totals that are biased upwards when weights are not calibrated.

A distance function has to be chosen for the calibration of the adjusted design-based weights. We explored only the linear distance function. When the sample is small, the linear approach may produce negative weights. While this was not an issue during this study, other methods, such as restricted calibration methods based on iterative algorithms, might be applied in such a case [14].

### Including the Sentinel-status

In scenarios without attractiveness heterogeneity, the RMSE decreases over an increasing number of cases (except for the weights adjusted for attractiveness heterogeneity). This is partly due to a decreasing number of non-reporting labs and thus a decrease in the number of labs wrongly classified as non-sentinel labs.

High weights are the result of few sentinel labs or low market shares. High weights can lead to unstable estimates. This problem can be solved by including more labs in sentinel surveillance, but if we are unable to recognize labs that are not reporting cases as sentinel labs simply including more labs will not be sufficient. Non-reporting, but participating labs are especially prevalent when there are only few cases to detect or when there is high detection heterogeneity between labs. In such scenarios the RMSE of the unadjusted and Huff-estimator increases as the number of sentinel labs increases.

For the unadjusted estimator, when there are only few sentinel labs, some provinces will not be taken into account. As the number of sentinel labs increases, more provinces will be included in the total and the estimate increases.

For the Huff-estimator another process is causing the increase in RMSE. Observed market shares will be either very high or zero, due to the simulated attractiveness heterogeneity. Small market shares are rarely observed. As we increase the number of sentinel labs the chance to include a small market share increases. Including small market shares will lead to coefficients that are closer to zero. With coefficients closer to zero the estimated market shares of non-reporting labs is higher. As these non-reporting labs are seen as non-sentinel labs, the market share of non-sentinel labs will increase, increasing the estimate.

## Abbreviations

IPW: Inverse Probability Weighting
labs: Laboratories
